# Assessing Undergraduates’ Perception of Risks Related to Body Art in Italy: The SUPeRBA Multicenter Cross-Sectional Study

**DOI:** 10.3390/ijerph18179233

**Published:** 2021-09-01

**Authors:** Carmela Protano, Federica Valeriani, Daniela Marotta, Annalisa Bargellini, Aida Bianco, Giuseppina Caggiano, Maria Eugenia Colucci, Maria Anna Coniglio, Laura Dallolio, Osvalda De Giglio, Gabriella Di Giuseppe, Pasqualina Laganà, Francesca Licata, Giorgio Liguori, Isabella Marchesi, Sofia Marini, Maria Teresa Montagna, Christian Napoli, Christian Napoli, Giovanni Battista Orsi, Cesira Pasquarella, Concetta Paola Pelullo, Vincenzo Romano Spica, Rossella Sacchetti, Stefano Tardivo, Licia Veronesi, Matteo Vitali, Francesca Gallè

**Affiliations:** 1 Department of Public Health and Infectious Diseases, Sapienza University of Rome, 00185 Rome, Italy; carmela.protano@uniroma1.it (C.P.); daniela.marotta@uniroma1.it (D.M.); giovanni.orsi@uniroma1.it (G.B.O.); 2 Department of Movement, Human, and Health Sciences, University of Rome “Foro Italico”, 00135 Rome, Italy; vincenzo.romanospica@uniroma4.it; 3 Department of Biomedical, Metabolic and Neural Sciences, Section of Public Health, University of Modena and Reggio Emilia, 41125 Modena, Italy; annalisa.bargellini@unimore.it (A.B.); isabella.marchesi@unimore.it (I.M.); 4 Department of Health Sciences, School of Medicine, University of Catanzaro “Magna Græcia”, 88100 Catanzaro, Italy; a.bianco@unicz.it (A.B.); francesca.licata@studenti.unicz.it (F.L.); 5 Department of Biomedical Sciences and Human Oncology, University of Bari “Aldo Moro”, 70124 Bari, Italy; giuseppina.caggiano@uniba.it (G.C.); osvalda.degiglio@uniba.it (O.D.G.); mariateresa.montagna@uniba.it (M.T.M.); 6 Department of Medicine and Surgery, University of Parma, 43125 Parma, Italy; mariaeugenia.colucci@unipr.it (M.E.C.); ira.pasquarella@unipr.it (C.P.); licia.veronesi@unipr.it (L.V.); 7 Department of Medical and Surgical Sciences and Advanced Technologies “G.F. Ingrassia”, University of Catania, 95123 Catania, Italy; ma.coniglio@unict.it; 8 Department of Biomedical and Neuromotor Sciences Alma Mater Studiorum, University of Bologna, 40126 Bologna, Italy; laura.dallolio@unibo.it (L.D.); sofia.marini2@unibo.it (S.M.); 9 Department of Experimental Medicine, University of Campania “Luigi Vanvitelli”, 80138 Naples, Italy; gabriella.digiuseppe@unicampania.it (G.D.G.); concettapaola.pelullo@unicampania.it (C.P.P.); 10 Department of Biomedical and Dental Sciences and Morphofunctional Imaging, University of Messina, 98122 Messina, Italy; plagana@unime.it; 11 Department of Movement Sciences and Wellbeing, University of Naples “Parthenope”, 80133 Naples, Italy; giorgio.liguori@uniparthenope.it (G.L.); francesca.galle@uniparthenope.it (F.G.); 12 Department of Medical Surgical Sciences and Translational Medicine, Sapienza University of Rome, 00189 Rome, Italy; christian.napoli@uniroma1.it; 13 Department of Computer Control and Management Engineering “Antonio Ruberti”, Sapienza University of Rome, 00185 Rome, Italy; cnapoli@diag.uniroma.it; 14 Department of Education Studies “Giovanni Maria Bertin”, University of Bologna, 40126 Bologna, Italy; rossella.sacchetti@unibo.it; 15 Department of Diagnostic and Public Health, University of Verona, 37134 Verona, Italy; stefano.tardivo@univr.it

**Keywords:** body art, piercing, tattooing, undergraduates, health, complications, knowledge, awareness

## Abstract

Tattooing and piercing may lead to health complications. The present multicenter cross-sectional study aimed to assess awareness and knowledge of health risks related to body art and to identify their possible determinants among a large sample of undergraduates in Italy. A web-based questionnaire collecting information on socio-demographic characteristics, awareness, knowledge, and some potential predictors was administered to undergraduates attending twelve Italian universities. The level of knowledge was expressed as the number of correct answers (0–11 for tattooing, 0–14 for piercing). A total of 2985 participants (mean age 23.15 ± 3.99, 73.9% F) participated in the study. Although 95.4% of the respondents were aware of possible health consequences of body art, a low level of specific knowledge was registered for both tattooing (mean number of correct answers 5.38 ± 2.39) and piercing (5.93 ± 3.12) consequences. Lower knowledge was associated with the attendance of non-life science course and with lower duration of academic education for both tattoo and piercing. Lower knowledge of tattooing risks was related with commuter status, while lower knowledge of piercing risks was associated with lower father’s education. These findings highlight the need to enhance information campaigns targeted to youths to increase their awareness of possible health risk of body art.

## 1. Introduction

In the last decades, body art practices such as tattooing and piercing have increased in popularity, becoming an accepted practice, and losing in people’s perception their traditional connection with deviant behaviors [1,2,3]. Although the exact prevalence of these practices is unknown, they have become mainstream in the USA and in Europe, mainly among adolescents and young adults [1,2,4].

Tattooing consists in the introduction of exogenous pigments into the dermis, resulting in a permanent design [3]. Tattoo ink may be made from several pigmented substances, including ashes, oils, and synthetic dyes. To make it available for fibroblast trapping, pigment is deposited to a depth of 1 to 2 mm into the dermis by using various instruments. In the traditional techniques, a sharp tool is used to cut the surface of the skin, and the pigment is pressed into the wound. Modern tattooing techniques employ motorized tattoo machines, which is less painful and allows for a more controlled application of ink, enabling the artist to produce more accurate designs and leading to minor bleeding [5].

Piercing is done by creating openings through the skin or cartilage to insert decorative ornaments such as rings, studs, or pins. Generally, body piercings are performed using a sharp, hollow needle designed for this purpose. The site to be pierced is usually held by a surgical clamp, through which a stainless-steel needle is pushed by hand into a cork or rubber stopper to create the hole. An open end of the jewelry is introduced into the rear blunt end of the needle and pulled through this opening, and the needle and stopper are removed. Modern body piercing jewelry contains substances such as stainless steel, titanium, gold, niobium, acrylic, or nickel, which may cause adverse reactions [1,5,6,7].

Both procedures imply the disruption of the skin/mucous barrier, allowing the penetration by endogenous or exogenous microorganisms in the underlying tissues and in the bloodstream and leading to local or systemic infections, if appropriate hygienic rules are not correctly followed. The risk and the severity of infection associated to these types of body modification depend on several factors, such as the body region involved, the employ of hygienic techniques during and after the procedure, the experience of the operator and the customer’s immune status. Besides, wound healing after tattooing or piercing depends on many factors, e.g., tissue characteristics (blood supply, cohesiveness), location and size of the puncture area, following aseptic procedures, reactivity of the human body to substances applied on/in the skin and deeper tissues [8]. In addition, allergic reactions to metals, inks, local anesthetic, and antiseptic cream employed during these procedures and other toxicity aspects of colorants have been reported in the literature [7,8,9,10,11].

In many countries, professional tattoo artists and piercers, who are licensed by local health departments, follow strict infection control techniques and procedures to eliminate the risk of microbial transmission during the body art application. They apply single use inks obtained from commercial vendors, sterilized needles, and disposable barriers to cover any parts of the equipment that may be exposed to body fluids [6]. However, tattoos/piercings applied in nonprofessional parlors, which are often made using household, non-sterile equipment, and improvised techniques, are still common throughout the world and may favor the development of complications [5,7]. Tattoos and piercings may be done in unregulated stores, jewelry shops, homes, by unlicensed personnel who have learned procedures from magazines, videos, or from other people. Furthermore, in the last years, a dangerous unregulated market of tattoo removal by cosmetologists, tattooists, nurses, and nonspecialized physicians has emerged, together with “do-it-yourself” tattoo removal procedures [12]. Some authors emphasized that the health risks in this service sector concern both clients and even professionals and therefore risk knowledge is fundamental to guarantee bilateral safety [12,13].

In this scenario, uninformed clients may not be aware of possible risks related to body art and are not able to identify licensed artists or to assess whether the tattooist/piercer is using proper procedures and equipment. Adolescents and young adults, who are particularly interested by this fashion, may undergo body art without informing their parents or in unauthorized facilities, showing a lack of perception of the possible health consequences of body art [2]. People who intend to have a tattoo/piercing should be informed about the risks of these practices, and in particular those with immune or skin disorders should discuss the procedure with their health care providers to take the appropriate precautions or avoid the procedure if necessary. In many countries the consent of legal guardians for minors under legal age who seek body modifications is required, as well as a medical consultation to avoid contraindications to these procedures, such as diabetes, hematological and neoplastic diseases, and immune system disorders, are needed [5,14,15].

Previous investigations performed to assess the awareness of health risks related to body art in Italian youths have highlighted some critical issues concerning their risk knowledge, with some geographical differences [16,17,18,19]. The present multicenter cross-sectional study was aimed at assessing the awareness and the knowledge of health risks related to body art practices in a large sample of undergraduate students from selected universities in Italy. The possible relationship between socio-demographic features and level of knowledge was also investigated.

## 2. Materials and Methods

The “Study on Undergraduate Perception of Risks of Body Art”—SUPeRBA—was carried-out between April 2020 and January 2021 involving undergraduate students from twelve universities selected by convenience throughout the whole Italian territory. The investigation was performed according to the principles embodied in the Declaration of Helsinki through a web-based questionnaire. Ethical approval was obtained from the Research Committee of the University of Rome “Foro Italico” (approval n CAR 31/2020) and from the academic deans.

### 2.1. Participants

Students attending the universities of Bologna, Modena and Reggio Emilia, Parma, Verona (northern Italy), Rome (central Italy), Bari, Catanzaro, Messina, and Naples (southern Italy) were invited to participate. The estimated total population included 400,971 undergraduates. A sample of at least 384 students would have been required, assuming a 95% confidence level and a 50% response proportion. A total of 3005 students (response rate 0.7%) completed the questionnaire. 

### 2.2. Questionnaire

A structured anonymous and voluntary questionnaire based on that used in our previous investigations was employed [17,18,19]. It included two sections. The first was focused on socio-demographic information: gender; age; university, year, and degree course attended; nationality, and educational level of parents. The second part was aimed at assessing the participants’ awareness about infectious and non-infectious risks associated with tattoos and piercing and methods of their removal. Multiple choices were made available to respondents. Briefly, for tattoo, 11 choices have been included: 5 infectious (viral hepatitis, bacterial infections, AIDS, tetanus, warts) and 6 non-infectious consequences (dissatisfaction, irritability, dermatitis, depression, allergy, scars). For piercing, 14 choices were possible: 5 infectious (viral hepatitis, bacterial infections, AIDS, tetanus, warts) and 9 non-infectious complications (dissatisfaction, gastritis, irritability, dermatitis, depression, prolonged bleeding, allergy, scars, choking). Furthermore, those who identified any of these risks were asked to indicate whether it is attributable to procedures, instruments, or environment. Participants were also asked to identify the removal procedure for tattoos (laser, peeling, cryotherapy, surgical removal, salt abrasion, injection of acid in the skin, or no removal procedure were available) and for piercing (surgery, natural closing of the tissue, or no removal procedure were available). The questionnaire and the aims of the study were presented to the undergraduates during lessons and administered through the Google modules platform. All the answers were coded and added in a database, specifically elaborated for statistical purposes.

### 2.3. Covariates and Statistical Analysis

A descriptive analysis was performed on sociodemographic characteristics and answers of participants. Continuous variables were expressed as mean values ± standard deviation (SD) while categorical variables were reported as number and percentage values of respondents. Considering the inclusion of health risks in the curriculum of the degree courses, participants were categorized as attending “life science” courses or not. The possible answers to the questions “Are there any health conditions that prevent having tattoos and/or piercings?”, “Can the tattoo procedure cause health problems?” and “Can the piercing procedure cause health problems?” were: “No”, “I do not know” and “Yes”. When the response to the last two questions was “Yes”, it was possible to choose which health problems were related to the tattoo/piercing procedure. The choices were divided in correct and wrong answers (coded as 1 and 0, respectively). The questions about the possibility of removing a tattoo or a piercing (“Is it possible to remove a tattoo?” and “Is it possible to remove a piercing?”) included the answers “No” (coded as 0) and “Yes” (coded as 1). When the response to the tattoo question was “Yes”, the method to remove a tattoo was investigated through the answers: “Laser”, “Chemical peeling”, “Cryotherapy”, “Salt abrasion”, “Surgical procedure”, and “Injection of acid in the skin”, coded with progressive numbers from 0 to 5. Those who responded “Yes” to the possibility to remove a piercing could choose the method between “Surgical procedure” (coded as 0) and “Spontaneous closure” (coded as 1). As for the level of knowledge, two variables were built by adding together for each participant the correct answers about tattooing (range 0–11) and piercing (range 0–14) and considering the median values for both of them: the resulting value was classified as “poor” when the sum of correct answers was lower than the median value, and “good” when the sum of correct answers was equal or higher than the median value.

Univariate analysis was performed to assess possible associations between variables and knowledge of tattooing/piercing health risks, using the chi squared test (with Yates’s correction). Finally, multivariate logistic regression was used to assess the possible association between knowledge of tattooing/piercing health risks and the variables examined (age, gender, university, course, year of study, residential status, father’s educational level, and mother’s educational level).

In order to perform univariate and multivariate analyses, the variables were codified as follows. Gender was expressed as female = 0 and male = 1 and the nationality as Italian = 0 and not Italian = 1; the universities attended by participants were grouped in the categories “North” (coded as 0) which included universities of Bologna, Modena and Reggio Emilia, Parma and Verona, “Center” (coded as 1) which included Rome universities and “South” (coded as 2) which includes universities of Bari, Catanzaro, Messina and Naples, and then grouped in the categories “North and Center” (coded as 0) and “South” (coded as 1) for the regression analysis; study courses were defined as “life science” (coded as 1) or “other” (coded as 0). For each student, the year of study was coded with the corresponding Arabic number from 1 to 6 (ex. First year of study coded as 1), with the answer “outside prescribed time” coded as 0, and then grouped in a dichotomous variable (≤3 years of study as 0 and >4 years of study as 1) for the logistic regression analysis; participants’ residential status was investigated through three options: “residing in the area” (coded as 0), “commuting” (coded as 1), and “not residing but living in the area” (coded as 2), and then coded as a dichotomous variable (“Residing or living in the university area” coded as 0 and “Commuting” coded as 1); parents’ educational level was codified as 0 for “Primary school”, 1 for “Middle school”, 2 for “High school”, and 4 for “Degree or post-degree”, and then aggregated as a dichotomous variable (“Primary/middle/high school” coded as 0 and “Degree or post-degree” coded as 1) for the logistic regression analysis. Adjusted odds ratio (OR) and 95% confidence intervals (CIs) were calculated. The significance level was assumed as *p* < 0.05. Analyses were conducted using the IBM SPSS Statistics for Windows, version 26.0 (IBM Corp., Armonk, NY, USA).

## 3. Results

The study population covered 400,971 students, of which 3005 subjects responded to the survey. Complete data from 2985 students were included in the analyses. Socio-demographic characteristics of the sample and answers related to tattoo and piercing health risks were summarized in Table 1.

Participants were mainly females, Italian, and attended life science courses. The most reported educational level of parents was the high school level. The 90.1% of the enrolled students knew that there are health conditions that prevent having tattoos and/or piercings. A great percentage (95.4%) declared that the tattoo procedure could cause health problems; similarly, 94.5% of the sample knew that piercing might induce them too. However, when asked to answer which health problems are linked to body art practices, an average of 5.38 ± 2.39 out of 11 correct answers was given for tattooing, while an average of 5.93 ± 3.12 out of 14 correct answers was found for piercing. More than half of the sample (60.5%) recognized that these health problems might be related with procedures, tools, and environments. Tattoos removal was possible for the 90.4% of the sample, and 92.5% identified laser as the correct procedure. Piercings can be eliminated according to 95.2% of the respondents and this can occur through spontaneous closure for 83.9% of them.

A univariate analysis was conducted to assess the possible association between the variables examined and the knowledge of health risks related to tattoos (Table 2).

Knowledge was dichotomized into “poor” and “good” levels referring to the median value (5) of correct answers related to the health consequences of tattooing. Age, degree course, year of study, residential status, father’s educational level, and mother’s educational level were significantly related with knowledge (Table 2).

Table 3 shows the univariate analysis performed on knowledge about health risks of piercing. Considering the median value (6) of correct answers to the question “which health risks are associated with piercing?”, knowledge was dichotomized as “good” (≥6) or “poor” (<6).

The variables age, university, course, year of study, residential status, father’s educational level, and mother’s educational level were significantly related with knowledge of piercing risk (Table 3).

Logistic regression models were built to identify variables independently associated with knowledge of health risks related to tattooing and piercing practices (Table 4).

Attending courses other than life science ones (OR 1.671, CI 95% 1.367–2.043) and attending university for 3 years or less (OR 1.941, CI 95% 1.582–2.382) were associated with a lower level of knowledge of tattooing health risks. Students residing and living in the university area were more likely to show good knowledge (OR 0.797, CI 95% 0.655–0.969).

Moreover, attending northern or central universities (OR 0.701, CI 95% 0.580–0.848) was associated with better knowledge of piercing health risks (Table 4). Participants who attended courses other than life science (OR 1.835, CI 95% 1.498–2.247) and universities for 3 years or less (OR 1.762, CI 95% 1.451–2.139), and whose fathers had elementary/middle/high school educational level (OR 1.291, CI 95% 1.056–1.577) showed lower knowledge of piercing consequences.

## 4. Discussion

### 4.1. Main Findings of This Study

This study was aimed at assessing the awareness of undergraduates from Italian universities about health risks related to body art. The findings show that almost all the respondents were aware of risks related to tattoo and piercing practice. However, a low level of specific knowledge was registered in the sample, in line with the insufficient knowledge on threats, contraindications, and complications related to tattoo and piercing practices of college students reported by previous studies [19,20,21,22,23].

Lower knowledge was associated with type of degree course and duration of attendance for tattoo and piercing risk. Knowledge of tattooing risks was also related with residential status, while knowledge of piercing risks was also associated with geographical area and father’s education.

### 4.2. What Is Already Known and What This Study Adds

In the comparison with the previous surveys performed in Italy, we learned about an increase in undergraduates’ risk awareness during the last decade. The studies previously performed in south Italy reported a general perception of risks related to tattoo and piercing ranging from 59.1 to 84.4% [16,17,18,19]. In the present study, despite the fact that students from southern universities showed a lower knowledge when compared with northern and center undergraduates, more than 94% declared their awareness towards tattoo and piercing health risks. However, in line with the previous experiences, participants showed a lower awareness regarding the type of health complications. In the analysis of the level of knowledge with respect to sociodemographic features, lower knowledge was reported by younger and commuting students, attending courses different from life sciences ones, attending southern universities and by those with lower parents’ educational levels. Among these variables, attending university for more than three years and attending life science courses were both associated in the regression analysis with higher knowledge of tattooing and piercing risk. This is in line with previous studies exploring the awareness of body art risks [23]. Furthermore, these findings are consistent with other studies concerning the knowledge about other health risks, such as that related to Severe Acute Respiratory Syndrome Coronavirus 2 (SARS-COV-2) infection during the pandemic of coronavirus disease 2019 (COVID-19). More precisely, a previous survey involving Albanian students showed a correlation between COVID-19 awareness and years of university education, while some studies performed among Italian undergraduates reported better knowledge of COVID-19 and better health literacy for life science/healthcare students than for students attending other degree courses [24,25,26,27].

As for the knowledge of tattooing risk, participants residing or commuting in the university area showed a higher level of knowledge than those living outside. A previous study performed in Italy underlined the difficulties that students who do not reside in the university area encounter in adopting a healthy lifestyle [27]. This may be due to the different engagement in living arrangement between these two categories of undergraduates, but also to different levels of health information, and requires further investigations. Attending universities from south Italy and having a father with a lower educational level were found to be associated with lower awareness of health risks related with piercing. The first aspect is noteworthy. With respect to this, it should be noted that all the universities included in the study belong to regions whose governments issued local regulations concerning body art practices in the last two decades. These documents establish the topics of the educational paths that tattooists and piercers must follow in order to respect hygienic rules during their activity and call on local health authorities to implement risk information campaigns for the general population. Several interventions have been performed accordingly in the corresponding Italian regions, even in southern regions. Our finding suggests the need to enhance these interventions, especially in South Italy.

As for the association between risk knowledge and father’s education, this is in line with another study performed among Indian adolescents [28].

Contrary to what was reported in previous Italian studies, age and having a tattoo or a piercing were not associated with the level of knowledge [16,17]. It is possible that these associations did not result from the analysis due to the narrow age range and the high proportion of non-tattooed and non-pierced individuals of the sample. In addition to this, evidence shows that psychosocial factors may play a role in determining the individual’s predisposition towards body modifications [29]. Although this study was not aimed at assessing psychosocial characteristics of participants, it is possible that their risk knowledge could have been related with their attitude and personal experience about tattoos/piercings. Further research is needed in this direction.

### 4.3. Limitations of This Study

This study has some limitations. Although the sample was built enrolling students from North, Center and South Italy, it was created by convenience and did not include students from all the Italian regions, and this hinders its representativeness. Considering that in Italy body art regulations are issued at regional level, it is possible that specific territorial differences were not detected. Moreover, many students came from life science faculties and courses whose curricula include notions of infection risk, pathogens, toxicology, and this could have biased the results related to risk knowledge. Furthermore, since only university students were enrolled, the results of the study cannot be extended to the whole Italian population of young adults. Finally, the sample showed a wide range of years of university education, and this could have influenced risk perception of participants. Further studies may be useful to highlight possible educational needs in this age class and/or to focalize on students attending specific academic years or courses. Possible differences in risk knowledge between youth who underwent tattoos/piercings and those who did not, together with possible psychosocial characteristics, should be also explored.

## 5. Conclusions

To conclude, although a high general perception of health risks related to body art was found among undergraduates in this study, poor specific knowledge about infective and non-infective complications was registered in students that had attended university for fewer years and those attending non-life science courses. Residential status, father’s education, and attending a university from South Italy were also identified as possible predictors of lower knowledge. These findings may contribute to addressing further risk information campaigns to specific groups of youths to increase their consumer awareness.

## Figures and Tables

**Table 1:** Characteristics of the sample and answers related to tattooing and body piercing (n = 2985).

Variables		n	%
Age (mean ± SD)	23.15 ± 3.99		
Gender	Female	2207	73.9
	Male	778	26.1
	Total	2985	
Nationality	Italian	2915	97.7
	Other	70	2.3
	Total	2985	
University	North	755	25.3
	Center	906	30.3
	South	1324	44.4
	Total	2985	
Course	Life science	2432	82.0
	Other	546	18.0
	Total	2978	
Year of study	First year	642	21.5
	Second year	682	22.8
	Third year	749	25.1
	Fourth year	156	5.2
	Fifth year	291	9.7
	Sixth year	266	8.9
	Outside prescribed time	199	6.7
	Total	2985	
Residential status	Residing in the area	873	29.2
	Commuting	1128	37.8
	Not residing but living in the area	984	33
	Total	2985	
Father’s educational level	Primary school	73	2.4
	Middle school	716	24.0
	High school	1404	47.0
	Degree or post-degree	792	26.5
	Total	2985	
Mother’s educational level	Primary school	74	2.5
	Middle school	562	18.8
	High school	1501	50.3
	Degree or post-degree	848	28.4
	Total	2985	
Do you have a tattoo?	No	2152	72.1
	Yes	833	27.9
	Total	2985	
Do you have a piercing?	No	1975	66.2
	Yes	1009	33.8
	Total	2984	
Are there any health conditions that prevent having tattoos and/or piercings?	No	84	2.8
	I do not know	211	7.1
	Yes	2690	90.1
	Total	2985	
Can the tattoo procedure cause health problems?	No	47	1.6
	I do not know	90	3.0
	Yes	2848	95.4
	Total	2985	
Health problems related to tattooing			
*Correct answers (mean ± SD)*	5.38 ± 2.39		
Can the piercing procedure cause health problems?	No	55	1.8
	I do not know	110	3.7
	Yes	2820	94.5
	Total	2985	
Health problems related to piercing			
*Correct answers (mean ± SD)*	5.93 ± 3.12		
What do you think these health problems are related to? (i) procedures, (ii) tools, and (iii) environments?	One of them	463	16.4
	Two of them	651	23.1
	All of them	1706	60.5
	Total	2820	
Is it possible to remove a tattoo?	No	287	9.6
	Yes	2698	90.4
	Total	2985	
How can a tattoo be removed?	Laser	2459	92.5
	Chemical Peeling	42	1.6
	Cryotherapy	6	0.2
	Salt abrasion	19	0.7
	Surgical procedure	118	4.4
	Injection of acid in the skin	14	0.5
	Total	2658	
Is it possible to remove a piercing?	No	144	4.8
	Yes	2841	95.2
	Total	2985	
How can a piercing be removed?	Surgical procedure	454	16.1
	Spontaneous closure	2358	83.9
	Total	2812	

**Table 2:** Univariate analysis of knowledge about tattooing health risks.

Variables		Poor Knowledge (n = 1070)	Good Knowledge (n = 1778)	*p*-Value
Age, mean ± SD		22.77 ± 3.93	23.41 ± 4.04	<0.001
Gender, n (%)	Female	803 (37.9)	1314 (62.1)	0.507
	Male	267 (36.5)	464(63.5)	
Nationality, n (%)	Italian	1043 (37.5)	1739 (62.5)	0.608
	Other	27 (40.9)	39 (59.1)	
University, n (%)	North	261 (36.6)	453 (63.4)	0.005
	Center	295 (33.9)	576 (66.1)	
	South	514 (40.7)	749 (59.3)	
Course, n (%)	Life science	809 (34.7)	1522 (65.3)	<0.001
	Other	256 (50.2)	254 (49.8)	
Year of study, n (%)	First year	253 (42.4)	343 (57.6)	<0.001
	Second year	320 (49.0)	333 (51.0)	
	Third year	269 (37.8)	442 (62.2)	
	Fourth year	58 (39.5)	89 (60.5)	
	Fifth year	71 (25.2)	211 (74.8)	
	Sixth year	48 (18.3)	215 (81.7)	
	Outside prescribed time	51 (26.0)	145 (74.0)	
Residential status, n (%)	Residing in the area	298 (35.8)	533 (64.1)	<0.001
	Commuting	471 (44.0)	599 (56.0)	
	Not residing but living in the area	301 (31.8)	646 (68.2)	
Father’s education level, n (%)	Primary school	36 (52.2)	33 (47.8)	<0.001
	Middle school	299 (43.8)	384 (56.2)	
	High school	503 (37.5)	840 (62.5)	
	Degree or post-degree	232 (30.8)	521 (69.2)	
Mother’s education level, n (%)	Primary school	32 (43.8)	41 (56.2)	<0.001
	Middle school	232 (43.6)	300 (56.4)	
	High school	544 (37.9)	891 (62.1)	
	Degree or post-degree	262 (32.4)	546 (67.6)	
Do you have a tattoo?	Yes	308 (39.2)	478 (60.8)	0.279
	No	762 (37.0)	1300 (63)	
Does your mother/father have a tattoo?	Yes	168 (40.3)	249 (59.7)	0.229
	No	902 (37.1)	1529 (62.9)	

**Table 3:** Univariate analysis of knowledge about piercing health risks.

Variables		Poor Knowledge (n = 1333)	Good Knowledge (n = 1487)	*p*-Value
Age, mean ± SD		22.95 ± 4.15	23.40 ± 3.88	<0.001
Gender, n (%)	Female	1002 (47.8)	1096 (52.2)	0.388
	Male	331 (45.8)	391 (54.2)	
Nationality, n (%)	Italian	1301 (47.3)	1450 (52.7)	0.903
	Other	32 (46.4)	37 (53.6)	
University, n (%)	North	309 (43.2)	406 (56.8)	0.001
	Center	388 (45.0)	475 (55.0)	
	South	636 (51.2)	606 (48.8)	
Course, n (%)	Life science	1015 (44.1)	1288 (55.9)	<0.001
	Other	313 (61.3)	198 (38.7)	
Year of study, n (%)	First year	318 (53.3)	279 (46.7)	<0.001
	Second year	366 (57.5)	270 (42.5)	
	Third year	329 (46.3)	382 (53.7)	
	Fourth year	68 (46.6)	78 (53.4)	
	Fifth year	112 (39.9)	169 (60.1)	
	Sixth year	69 (26.4)	192 (73.6)	
	Outside prescribed time	71 (37.8)	117 (62.2)	
Residential status, n (%)	Residing in the area	385 (47.0)	435 (53.0)	0.001
	Commuting	546 (51.3)	519 (48.7)	
	Not residing but living in the area	402 (43.0)	533 (57.0)	
Father’s education level, n (%)	Primary school	39 (57.4)	29 (42.6)	<0.001
	Middle school	367 (55.5)	294 (44.5)	
	High school	624 (46.8)	709 (53.2)	
	Degree or post-degree	303 (40.0)	455 (60.0)	
Mother’s education level, n (%)	Primary school	42 (58.3)	30 (41.7)	0.001
	Middle school	277 (53.5)	241 (46.5)	
	High school	664 (46.9)	752 (53.1)	
	Degree or post-degree	350 (43.0)	464 (57.0)	
Do you have a piercing?	Yes	446 (47.0)	502 (53.0)	0.873
	No	887 (47.4)	984 (52.6)	
Does your mother/father have a piercing?	Yes	75 (47.8)	82 (52.2)	0.935

**Table 4:** Logistic regression analysis of variables related to poor knowledge of tattooing and piercing risks.

Variables	%	Tattooing Risk	Piercing Risk
		Adjusted Odds Ratio (CI 95%)	Adjusted Odds Ratio (CI 95%)
Age		0.992 (0.971–1.014)	0.999 (0.978–1.020)
Gender			
- Female	73.9	0.947 (0.790–1.135)	0.952 (0.798–1.135)
- Male	26.1	Reference	Reference
University			
- North and Center	55.6	0.824 (0.678–1.002)	0.701 (0.580–0.848) **
- South	44.4	Reference	Reference
Course of study			
- Other	18.0	1.671 (1.367–2.043) **	1.835 (1.498–2.247) **
- Life Science	82.0	Reference	Reference
Year of study			
- ≤3 years	69.4	1.941 (1.582–2.382) **	1.762 (1.451–2.139) **
- >4 years	30.6	Reference	Reference
Residential status			
- Residing and living in the area	62.2	0.797 (0.655–0.969) *	0.956 (0.789–1.158)
- Commuting	37.8	Reference	Reference
Father’s education level			
- Primary/middle school/high school	73.5	1.177 (0.953–1.453)	1.291 (1.056–1.577) *
- Degree or post degree	26.5	Reference	Reference
Mother’s education level			
- Primary/middle school/high school	71.6	1.084 (0.884–1.329)	0.984 (0.810–1.196)
- Degree or post degree	28.4	Reference	Reference

* *p* < 0.05; ** *p* < 0.001.

## Data Availability

Not applicable.
